# Somatic neural alterations in non-diabetic obesity: a cross-sectional study

**DOI:** 10.1186/s40608-016-0131-3

**Published:** 2016-11-22

**Authors:** Ram Lochan Yadav, Deepak Sharma, Prakash Kumar Yadav, Dev Kumar Shah, Kopila Agrawal, Rita Khadka, Md. Nazrul Islam

**Affiliations:** 1 Department of Physiology, Chitwan Medical College, Bharatpur, Nepal; 2 Department of Physiology, BP Koirala Institute of Health Sciences, Dharan, Nepal

**Keywords:** Non-diabetic obesity, Nerve conduction study, BMI, Somatic neuronal functions

## Abstract

**Background:**

Reports on alterations in somatic neural functions due to non-diabetic obesity, a major risk factor for diabetes, are few and still a matter of debate. Nevertheless, to our knowledge, reports lack any comments on the type of somatic nerve fibers affected in non-diabetic obesity. Therefore, this study aimed to find out the alteration in somatic neural functions in non-diabetic obese persons if any.

**Methods:**

The study was conducted on 30 adult non-diabetic obese persons (mean age 32.07 ± 7.25 years) with BMI > 30 Kg/m^2^ (mean BMI 30.02 ± 2.89 Kg/m^2^) and 29 age- and sex-matched normal weight controls (mean age 30.48 ± 8.01 years) with BMI: 18–24Kg/m^2^ (mean BMI 21.87 ± 2.40 Kg/m^2^). Nerve conduction study (NCS) variables of median, tibial and sural nerves were assessed in each subject using standard protocol. The data were compared by Mann Whitney ‘U’ test.

**Results:**

In comparison to normal weight persons, obese had lower compound muscle action potential (CMAP) amplitudes of right median [9.09(7.62–10.20) Vs 10.75(8.71–12.2) mV, *p* = 0.025] and bilateral tibial nerves [Right: 8.5(7.04–11.18) Vs 12.1(10.55–15) mV, *p* < 0.001 and left 9.08(6.58–11.65) Vs 13.05(10.2–15.6) mV, *p* = 0.002]. Furthermore, obese persons had prolonged CMAP durations of right and left median [10.5(9.62–12) Vs 10(8.4–10.3) ms, *p* = 0.02 and 10.85(10–11.88) Vs 10(9–10.57) ms, *p* = 0.019] and right tibial [10(9–11) 8.5(7.92–10) ms, *p* = 0.032] nerves. Sensory NCS (sural nerve) also showed diminished sensory nerve action potential (SNAP) amplitude [16(12.08–18.21) vs 22.8(18.3–31.08) μV, *p* < 0.001] and prolonged duration. However, onset latencies and conduction velocities for all nerves were comparable between the groups.

**Conclusion:**

This study documents subclinical peripheral nerve damage in non-diabetic obese with abnormal NCS parameters; shorter amplitudes and prolonged CMAP and SNAP durations. The reduced amplitudes of mixed and sensory nerves might be due to decreased axonal number stimulation or actual decrease in number of axonal fibers, or defect at NMJ in non-diabetic obese. Prolonged durations but normal onset latencies and conduction velocities strongly suggest involvement of slow conducting fibers.

## Background

Obesity is a medical condition with a multifactorial etiology. Due to worldwide prevalence of this disorder, it is increasingly considered one amongst the major public health problems, with at least 2.8 million people dying each year as a result of being overweight or obese. Moreover, obesity has become common place in many low- and middle-income south Asian countries, including Nepal and Bangladesh with prevalence being 10% and 8.9% respectively. There are many reports suggesting that obesity causes various alterations in hemodynamic and metabolic systems which affect the functions of many organs and the systems [1, 2].

Obesity is a major risk factor for diabetes. There are substantial reports and established findings on alterations in somatic neural functions due to diabetes [3]. However, only a few have reported functional alterations in somatic neurons merely due to obesity [4]. Further in our geographical setting, reports lack commenting on somatic nerve functions in non-diabetic obese. Therefore, the effects of obesity on somatic neuronal functions are controversial and need to be explored. To our knowledge, reports lack any comments on the type of somatic neural fibers affected most while alterations in somatic neural functions due to non-diabetic obesity.

Somatic sensory and motor nerve conduction studies (NCS) were used to assess their structural and functional alterations [5, 6]. Thus, the study aimed to investigate motor (MNCS) and sensory (SNCS) nerve conduction in non-diabetic obese patients to examine the relevance for the development of subclinical peripheral nerve damage along with exploring the possible order or type of neural fibers involvement. Further, study can be extended to investigate the cause for alterations in neural functions, if any, which may be due to metabolic or structural alterations in neurons as a result of obesity.

## Methods

### Subjects

This cross-sectional comparative study was conducted over a year (2011–2012) in the Neurophysiology laboratory in the Department of Basic and Clinical Physiology, B. P. Koirala Institute of Health Sciences (BPKIHS), Dharan, Nepal. The convenient sampling was used for the recruitment of the subjects and was selected purposively among the medical staffs and students at BPKIHS having similar lifestyle to avoid the effect of amount, type and intensity of physical activity on nerve response. Thirty non-diabetic obese individuals (Age 32.07 ± 7.25 years) and 29 age- matched normal weight controls (Age 30.48 ± 8.01 years) participated in the study. The percentage contribution of male and female was 50% each in obese group, and 53.33% male and 46.66% female was in normal weight group.

Mean BMI of the obese patients and controls were (32.02 ± 2.89 Kg/m^2^) and (21.87 ± 2.40 Kg/m^2^), respectively, *p* < 0.001. Obese was defined (WHO) as a BMI of over 30, and normal weight was defined as a BMI of less than 25, i.e., between 18 and 24(kg/m^2^) [7, 8]. To be included, subjects were required to be between 18 and 75 years old and they also had to meet the BMI criteria noted above. Informed written consent was taken from all the subjects and they were screened for any history of drugs/alcohol intake, upper and lower extremity fractures, the familial history of neuropathy, or medical illness likely to affect the nerve conduction study parameters based on clinical history and physical examinations including detailed neurological assessment. Diabetic persons were excluded from the study according to American Diabetes Association criteria (American Diabetes Association, 2003), which defines a plasmatic basal glucose level higher than 126 mg/dL as a reliable indicator of diabetes [9]. The choice of the patients was very selective, to attribute a potential pathogenetic value to a metabolic alteration typical of the obese patients, represented by normal blood glucose level. Plasma glucose level was measured in the clinical laboratory, Department of Biochemistry, BPKIHS, Dharan, Nepal. This study was conducted according to the guidelines of the Declaration of Helsinki and approved by the ethical committee, BP Koirala Institute of Health Sciences, Dharan, Nepal.

Room temperature of the laboratory was maintained at the thermo neutral zone i.e., 26 ± 2 °C. All the required set up was checked before commencing the test. Further, subjects were made comfortable and familiar with the laboratory set up and conditions, and were advised to relax completely during recording. A conventional neurographic study was performed to measure the NCS parameters under standard laboratory conditions by using a Nihon Kohden machine (NM-420S; H36, Japan).

## Recording procedures

### Anthropometric and blood pressure measurement

Age was recorded in complete years i.e., rounded of the nearest years. Standing height was recorded with stadiometer (Prestige brand) by making the subject stand without shoes and shocks with feet parallel and pointing forwards on a special platform to which vertical measuring rod, marked in metric scale was fixed. Subject was ensured to stand as tall as possible with positioning head in the Frankfort plane position (the plane joining lower border of left orbit and the upper margin of the external auditory meatus in horizontal position). The head piece of the stadiometer or the sliding part of the measuring rod was lowered so that the hair (if present) was pressed flat. Height was recorded in cm to the resolution of the height rule (i.e., the nearest quarter of a cm). Weight of each subject was taken on balanced beam scale (Dr. Morepen MS02B Mechanical weighing machine). The scale was placed on hard floor surface and calibrated with standardized weights at the beginning and end of each examining day. The subjects after removing their heavy outer garments (jacket, coat, trousers, skirts, wrist watch, mobile from pocket etc.) were asked to stand in the centre of the platform to distribute body weight evenly to both feet. The weight was recorded to resolution of the scale (the nearest 0.1 kg to 0.2 kg). Body mass index (BMI) was calculated from the standard formula i.e., BMI = weight (kg)/height (m^2^). Blood pressure (BP) was measured manually using mechanical aneroid sphygmomanometer and a quality stethoscope (MDF ® 808 Professional Aneroid Sphygmomanometer and Stethoscope). Systolic and Diastolic blood pressure were recorded in sitting upright position after a minimum of five-minute rest, palpatory was followed by ascultatory method of blood pressure measurement. BP was double checked for accuracy as recommended by AHA (American Heart Association) by taking reading with both arms and averaging the readings. Three readings were taken in the interval of 5 min rest and the average of readings was taken as final systolic and diastolic blood pressures.

### Nerve conduction study

The neurophysiological study consisted of motor NCS (MNCS) and sensory NCS (SNCS) in median (mixed), tibial (motor), and sural (sensory) nerves. Nerve conduction studies were performed using standard techniques of supra-maximal percutaneous stimulation with a constant current stimulator and surface recording electrodes of electromyography on both extremities of each subject. SNCS was performed orthodromically for median nerves, anti-dromically for the sural nerves, using disposable surface electrodes with supramaximal stimulation. Skin surface temperature was measured over the dorsum of the hand and foot. The limb was warmed with a hairdryer if temperature was below 32 °C. Filter settings were 2 Hz to 10 kHz and 20 Hz to 2 kHz, respectively, for motor and sensory recordings.

i) Motor nerve conduction study variables
  For motor nerve conduction study, the stimulator with water soaked felt tips were used for surface stimulation. It was placed on the skin overlying the nerve at two or more sites (Table 1) along the course of nerve after cleaning the site with skin purifier. Before applying a brief pulse of current, ground electrode was placed between the stimulating and recording electrodes. The recording and the reference electrodes were placed using belly tendon montage with the recording electrode placed over the mid belly of the respective muscles, as close to the estimated end plate site as possible and the reference electrode to the tendon at a minimum distance of 3 cm. The sites of stimulation and recording electrodes for different nerves tested are shown in the Table 1 as described by Aminoff [5] and Misuilis [6].

**Table 1:** Stimulation and recording sites of motor nerves

Motor Nerve	Site of stimulations		Recording site (muscle)
	Proximal site	Distal site	
Median Nerve	Antecubital fossa	Wrist	Abductor pollicis brevis
Tibial Nerve	Popliteal fossa	Medial ankle	Abductor Hallucis brevis
  Burst of direct current with supramaximal stimulus and the electrical pulse duration of 200 μsec were used for the stimulation of nerve being studied. While stimulating, cathode of stimulator was faced distally. For each stimulation site (distal and proximal), latency, amplitude, duration and NCV were measured from the obtained CMAP.
  Once a recording was made, the trace was stored for later analysis and the stimulating electrode was moved proximally to the second site. The distance between stimulating electrodes at proximal and distal sites was measured with a measuring tape and was fed into the machine for nerve conduction velocity calculation. For each stimulation site, onset latency was measured in milliseconds from the stimulus artefact to the first deflection of CMAP whereas peak latency was measured at the midpoint of the first negative peak. CMAP amplitude was measured from baseline to the negative peak (base to peak) and duration of CMAP was measured from the onset to the final return of waveform to the baseline (Fig. 1). For recording F-response or the late response, the stimulator was placed at the distal point of stimulation of each nerve with cathode proximally. Maximum, minimum and mean F-wave latencies were then measured (Fig. 2).

**Fig. 1:**
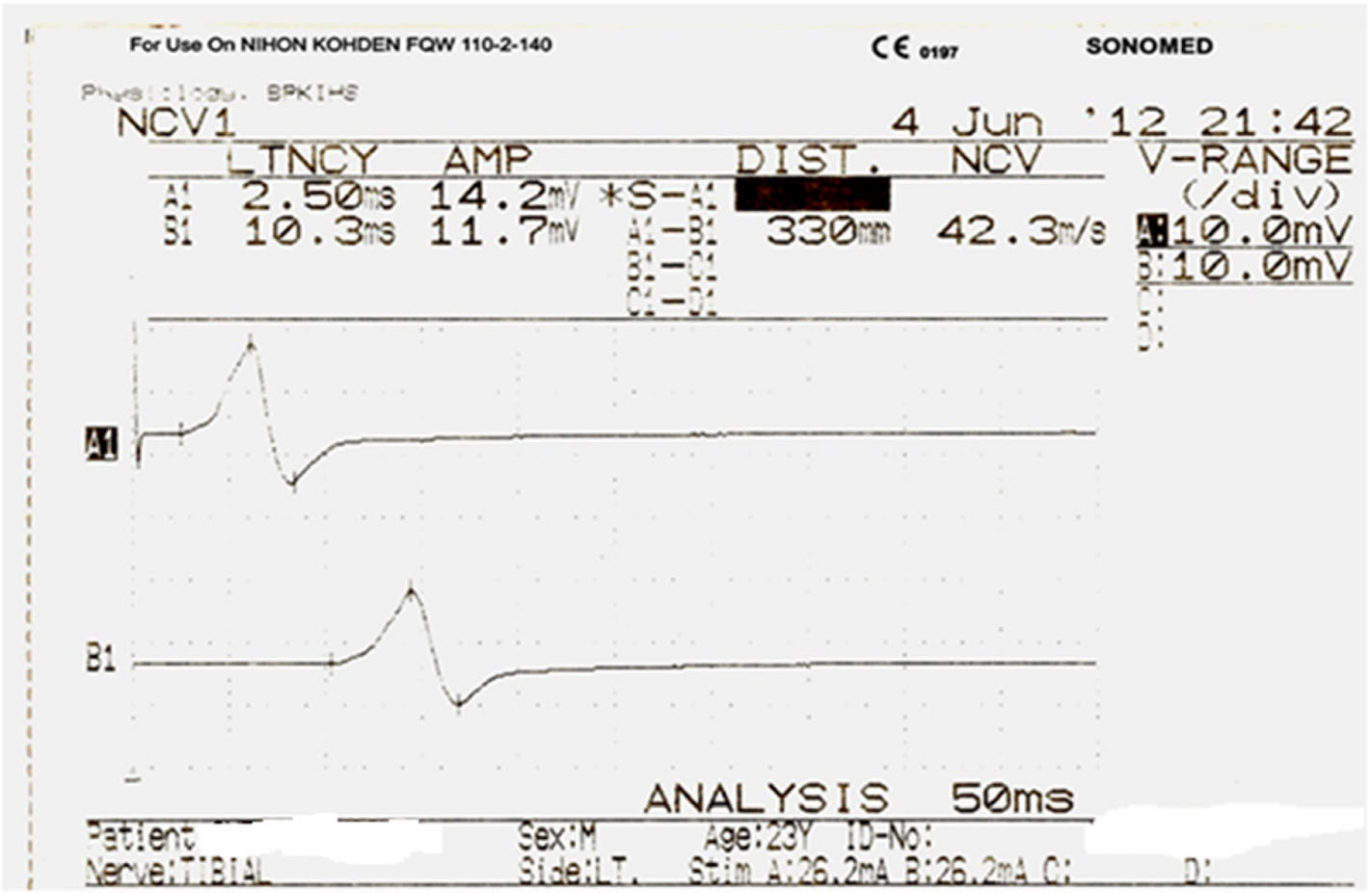
Representative trace of tibial distal and proximal compound muscle action potential

**Fig. 2:**
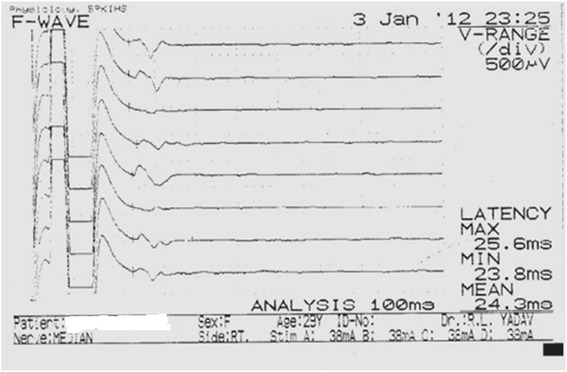
F-wave for Median nerve
ii) Sensory nerve conduction study variables
  For sensory nerve conduction study, orthodromic and antidromic method of stimulation was employed. Gain was set at 10 mV per division. The electrodes were essentially the same as those used for motor NCV. Skin purifier (skin pure) was used to clean electrode (recording) sites and to reduce skin surface impedance. Stimulating or recording electrodes were placed on a purely sensory portion of the nerve. Before stimulating the nerve, ground electrode was placed between the stimulating and recording electrodes. Antidromic method of stimulation was used for sural nerve and orthodromic used for median nerve. The sites of stimulation and recording electrodes for different nerves are shown in the Table 2 as described by Aminoff [5] and Misuilis [6]. For orthodromic conduction, ring electrodes were used to stimulate the digital nerve whereas surface stimulating electrodes were used for antidromic stimulation. An electrical pulse of either 0.1 m second duration was used and most nerve required a current in the range of 16 to 30 mA to achieve supra maximal stimulation. Current was slowly increased from a base liner of 0 mA, usually by 3–5 mA at a time until the recorded sensory potential was maximized. For each nerve, latency, amplitude, and conduction velocity of SNAP were recorded. Onset latency was measured from stimulus to the onset of initial negative deflection for biphasic SNAPs and to the initial peak for triphasic SNAPs. Peak latency was measured at the midpoint of the first negative peak. For this study the average of twenty responses was taken. SNAPs are usually biphasic or triphasic potentials. Unlike in motor studies, in sensory studies, conduction velocity was calculated with single stimulation because there is no transmission along neuromuscular junctions (NMJ) or muscle fibre. The duration of SNAP was measured firstly from the initial negative peak to return to the baseline. SNAP amplitude was measured form the baseline to negative peak.

**Table 2:** Stimulation and recording sites of sensory nerves

Sensory nerve	Method of stimulation	Stimulation sites	Recording sites
Sural	Antidromic	Posterior-lateral calf	Posterior ankle
Median	Orthodromic	Index finger	Middle of the wrist

### Statistical analysis

The SPSS software (v 16.02, Chicago, Illinois, USA) for personal computer was used for the statistical analyses. Shapiro-Wilk’s W test was applied to examine normality in the distribution of data. Anthropometric, cardiorespiratory and biochemical observations were distributed normally, therefore, parametric student’s *t*-test was applied for the statistical comparison and data were expressed in mean ± SD. Since the remaining observations had non-parametric distributions, the Mann whitney ‘U’ test was applied for the statistical comparison between study groups. The data were expressed as median (inter-quartile range) in tabulated form and *p* < 0.05 was the limit for a significant difference.

## Results

### Anthropometric and cardiorespiratory variables

Among the studied variables between the groups weight, body mass index, pulse rate, systolic blood pressure, and diastolic blood pressure were found to be significantly more in obese groups shown in Table 3.

**Table 3:** Comparison of anthropometric, cardiorespiratory and biochemical variables between obese (*n* = 30) and normal weight (*n* = 29) groups

Variables	Normal weight (*n* = 29) Mean ± SD	Obese (*n* = 30) Mean ± SD	*P* value
Age, yrs	30.48 ± 8.012	32.07 ± 7.25	0.429
Height, m	1.66 ± 0.10	1.60 ± 0.099	0.038
Weight, kg	60.69 ± 9.43	82.93 ± 11.14	**<0.001**
BMI, kg/m^2^	21.87 ± 2.40	30.02 ± 2.89	**<0.001**
Respiratory rate, cycles/min	16.41 ± 2.87	16.60 ± 2.76	0.801
Pulse rate, beats/min	71.48 ± 8.41	79.17 ± 8.80	**<0.001**
Systolic blood pressure, mmHg	113.24 ± 11.07	121.20 ± 9.89	**0.005**
Diastolic blood pressure, mmHg	74.83 ± 10.31	84.97 ± 7.87	**<0.001**
Fasting blood sugar, mg/dl	85.72 ± 15.36	84.80 ± 17.52	0.830

The bold values in the tables shows the data are statistically significant

### Nerve conduction study (NCS) variables

**Fig. 3:**
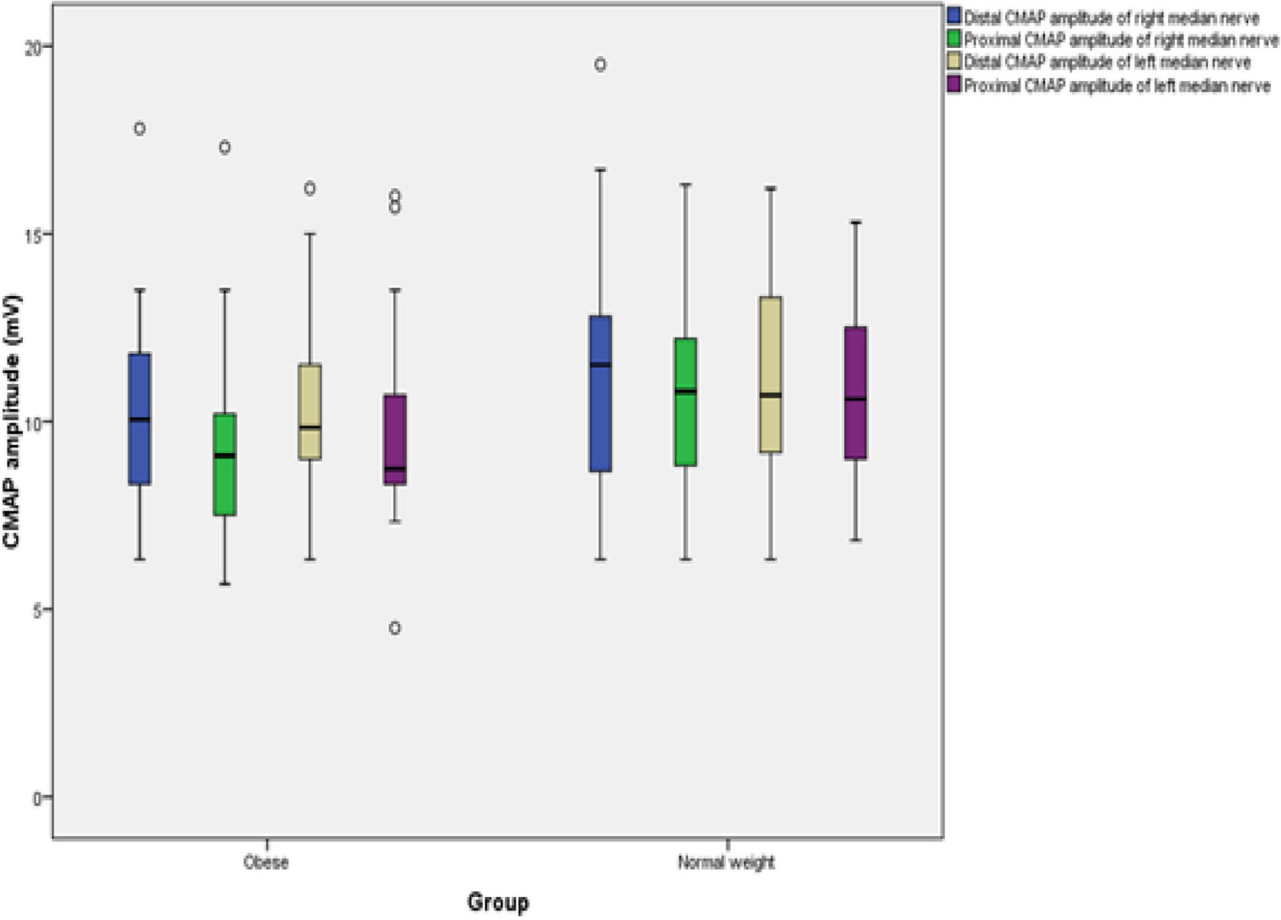
CMAP amplitudes of median nerve in non-diabetic obese and normal weight adults

**Fig. 4:**
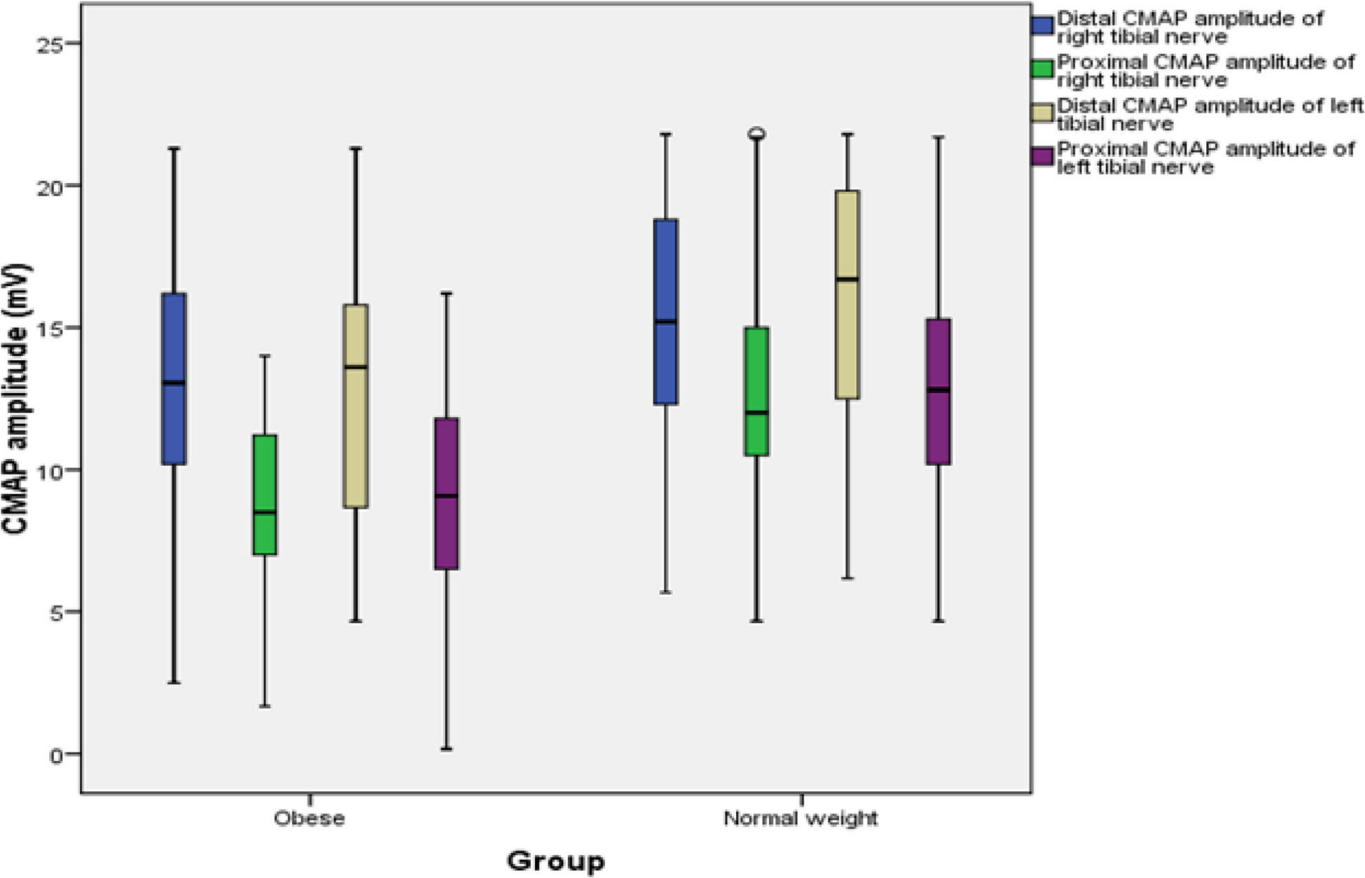
CMAP amplitudes of tibial nerve in non-diabetic obese and normal weight adults

**Fig. 5:**
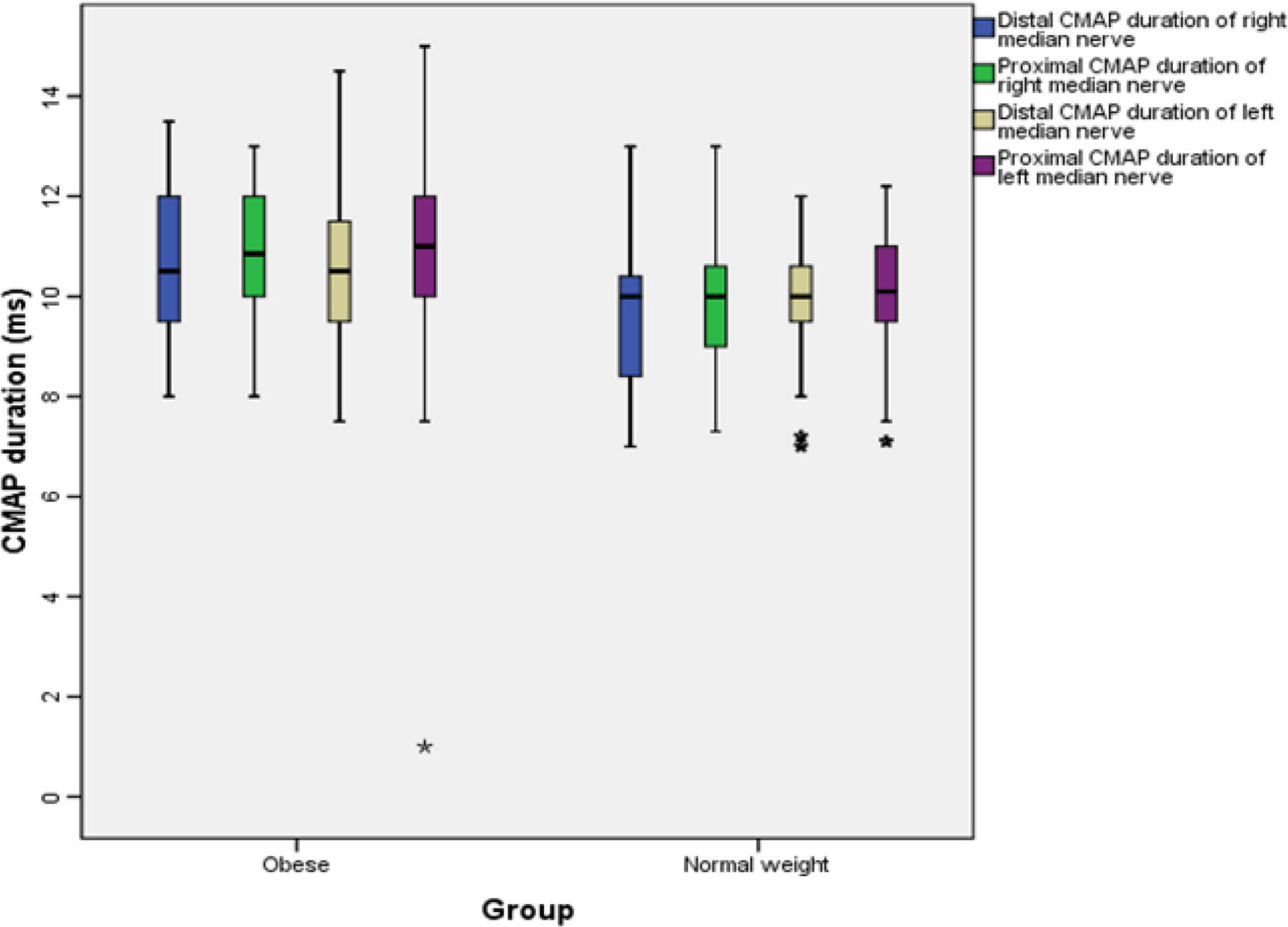
CMAP durations of median nerve in non-diabetic obese and normal weight adults

**Fig. 6:**
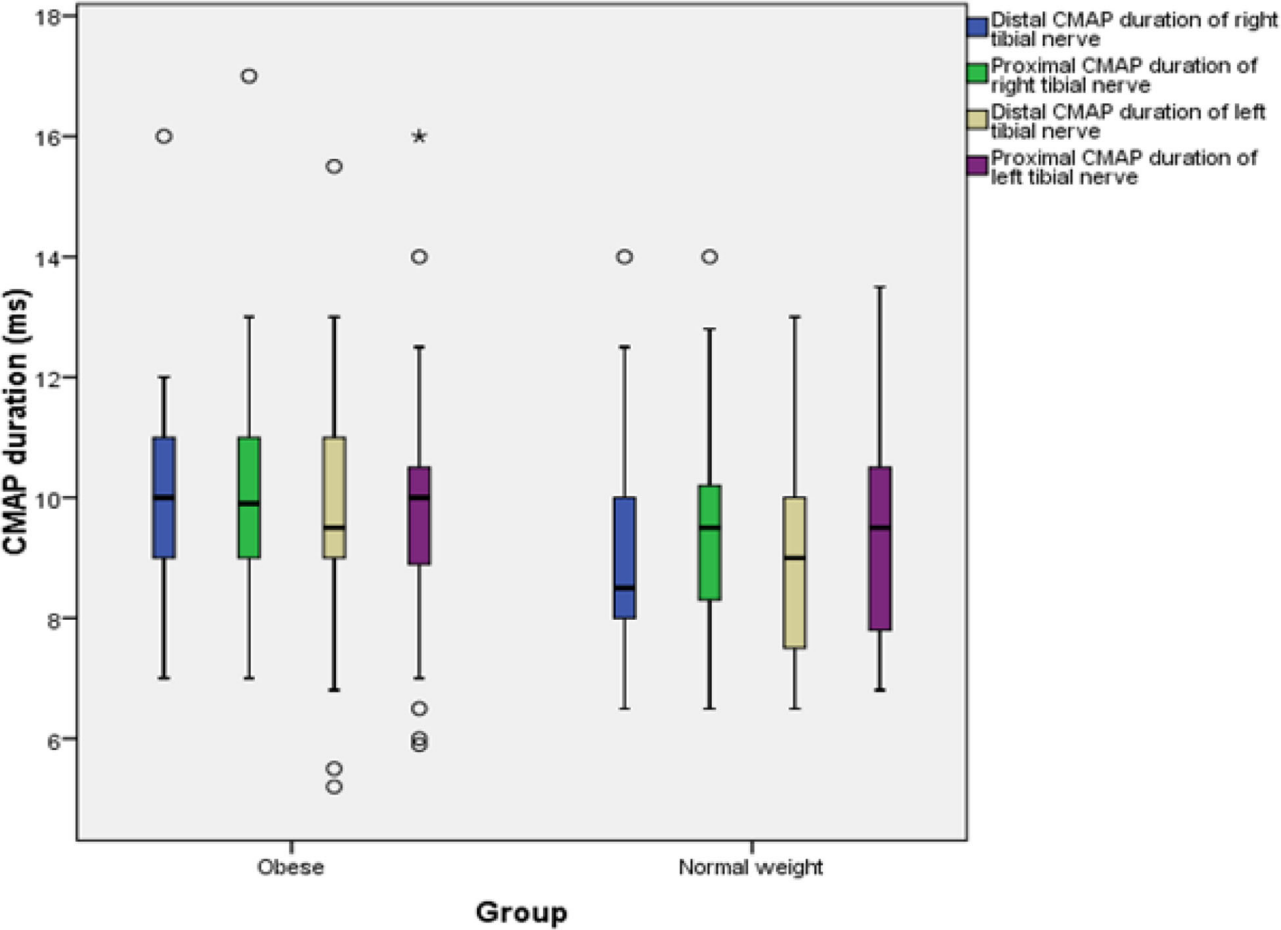
CMAP durations of tibial nerve in non-diabetic obese and normal weight adults

**Fig. 7:**
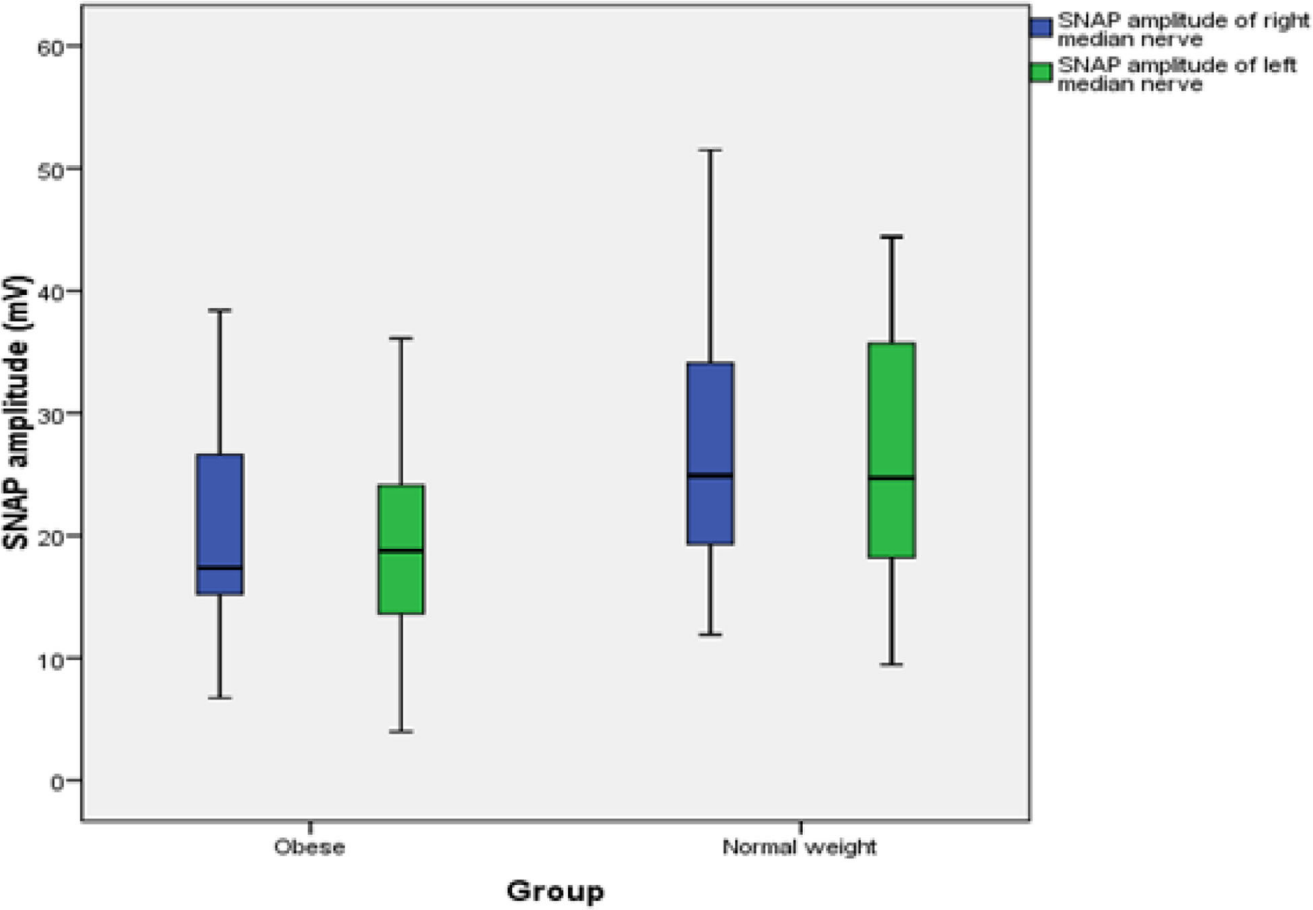
SNAP amplitude of median nerve in non-diabetic obese and normal weight adults

**Fig. 8:**
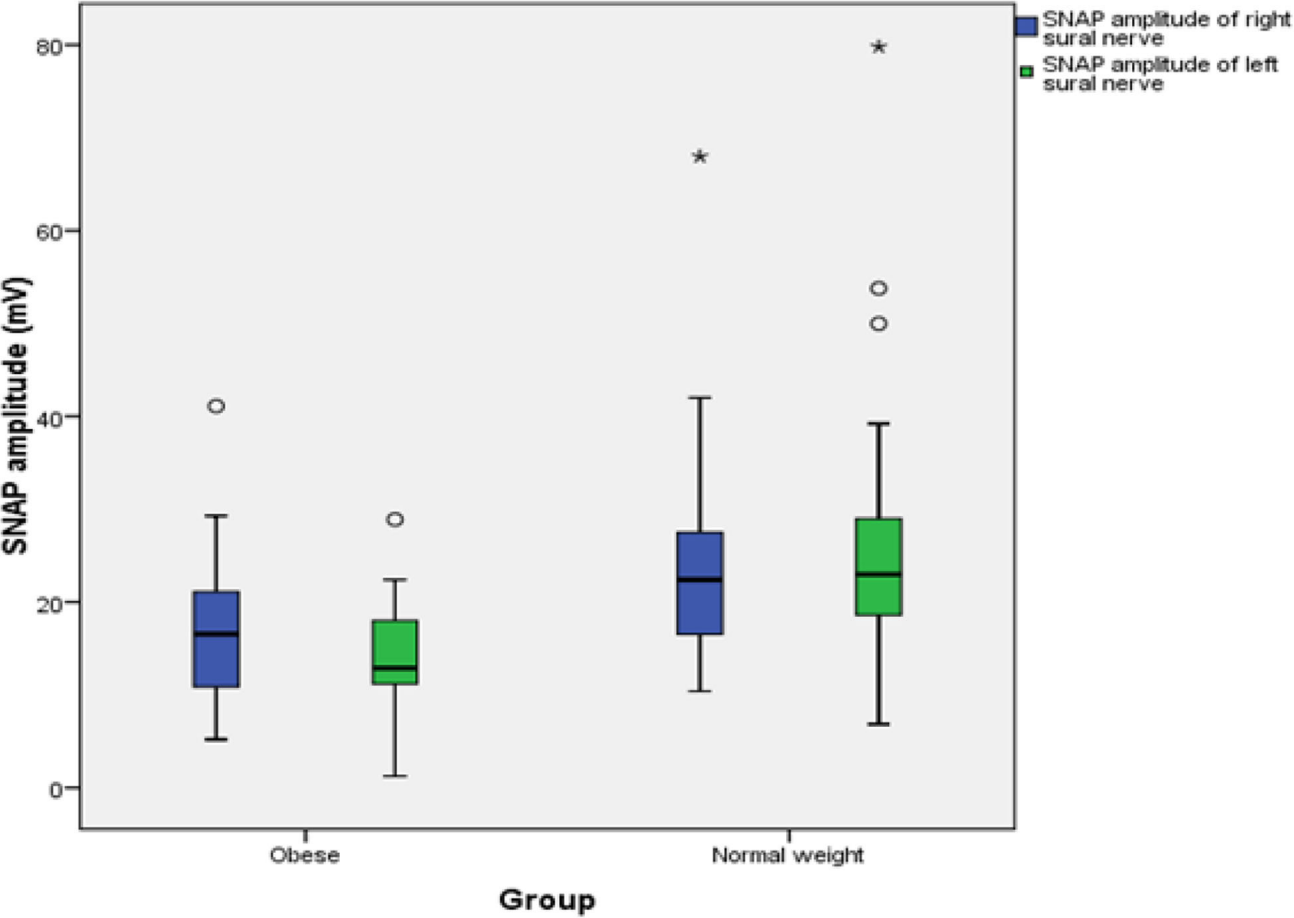
SNAP amplitude of sural nerve in non-diabetic obese and normal weight adults

**Fig. 9:**
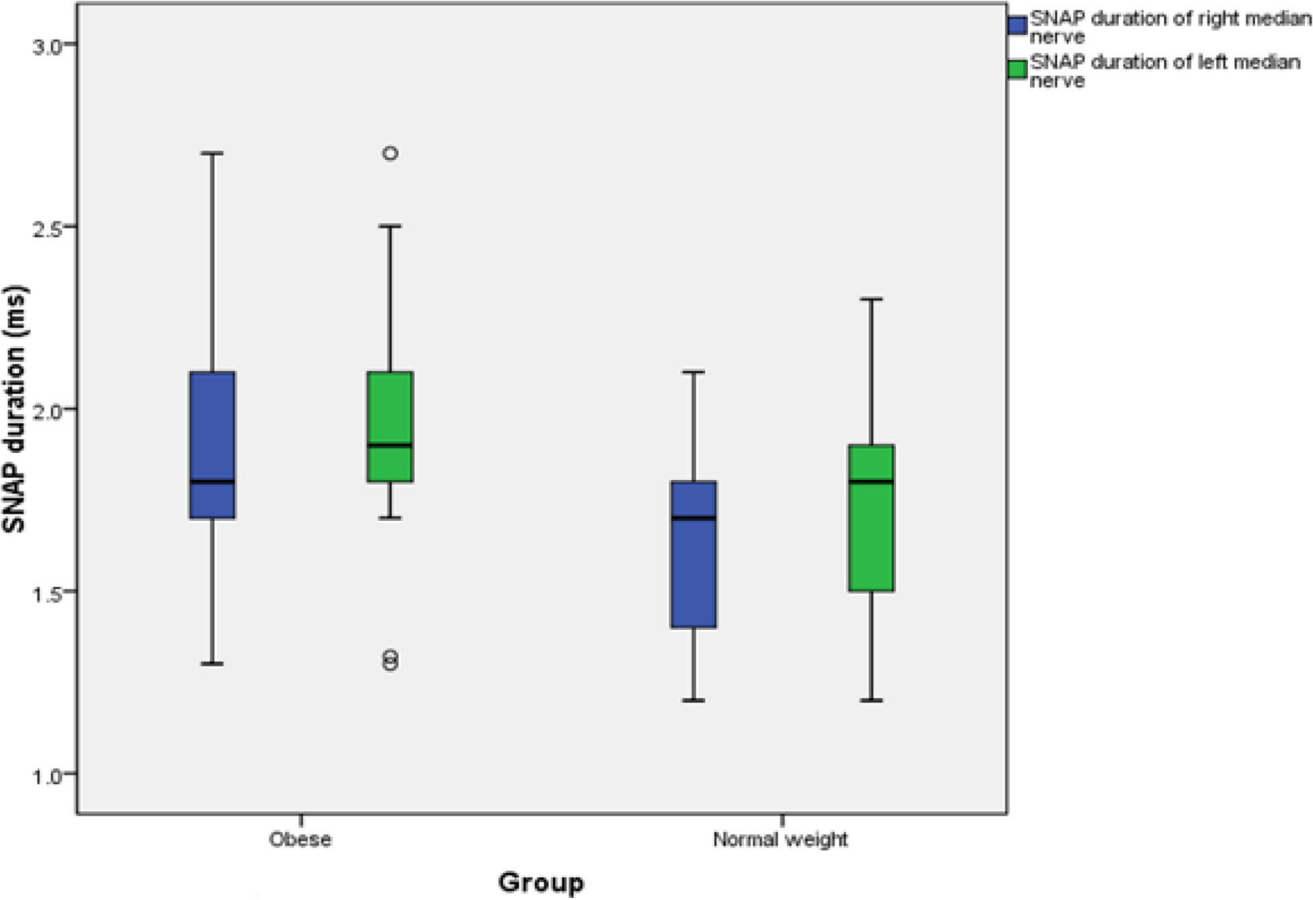
SNAP duration of median nerve in non-diabetic obese and normal weight adults

**Fig. 10:**
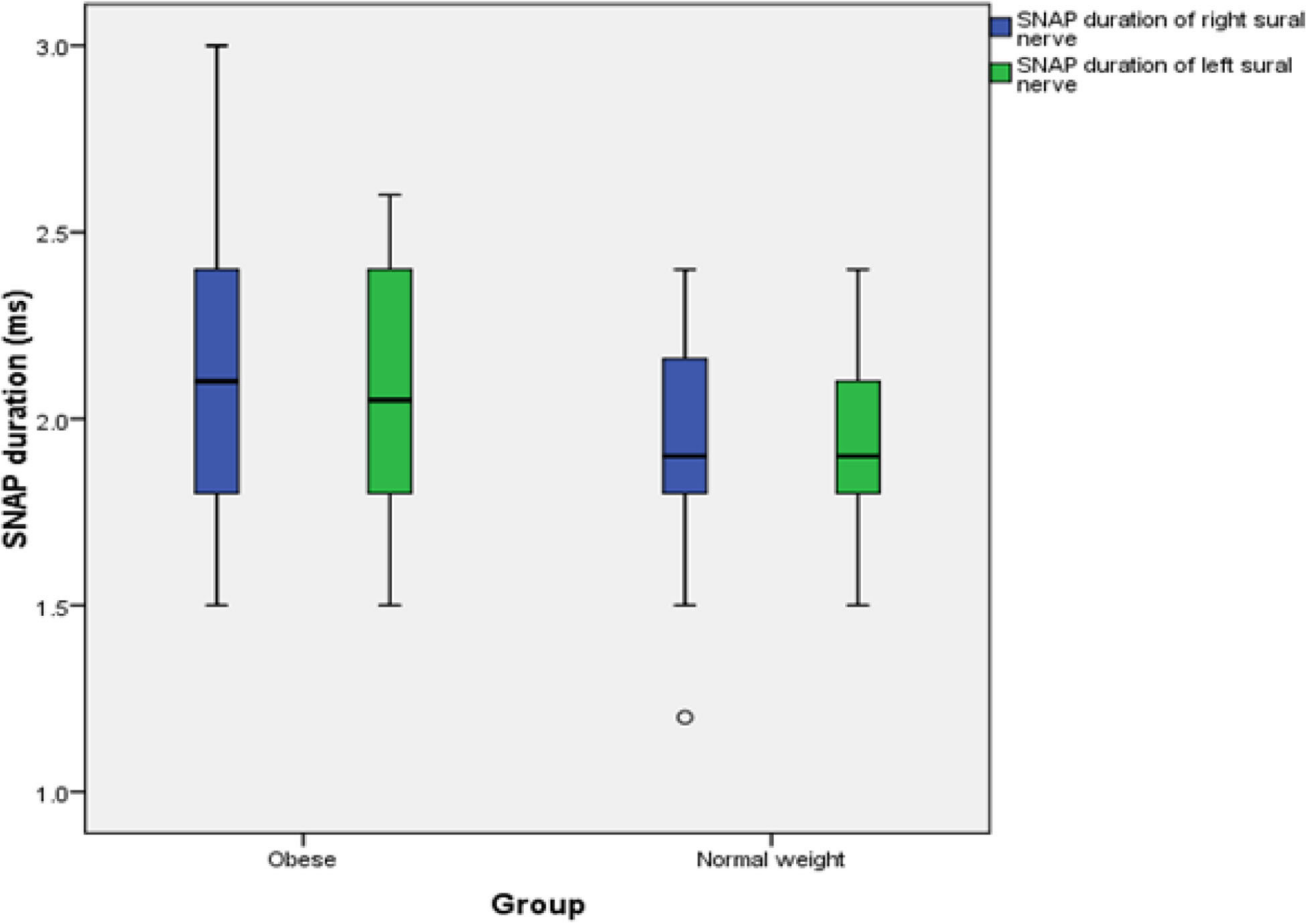
SNAP duration of sural nerve in non-diabetic obese and normal weight adults

The findings for MNCS and SNCS of obese and normal weight groups are presented in Tables 4, 5, 6 and 7. Among the studied motor NCS variables, compound muscle action potential (CMAP) amplitudes of right median (Table 4 and Fig. 3), right and left tibial (Table 5 and Fig. 4) nerves were significantly less in obese compared to control group. Distal and proximal CMAP durations for right median (Fig. 5) and tibial nerves (Fig. 6) of both sides were significantly prolonged in obese. The findings for sensory NCS were similar to that of motor NCS. The sensory nerve action potential (SNAP) amplitudes of median (Table 6 and Fig. 7) and sural nerves of both sides (Table. 7 and Fig. 8) were found to be reduced whereas SNAP durations for them (Figs. 9 and 10) were prolonged significantly in obese than in normal weight group.

**Table 4:** Comparison of median motor nerve conduction study (NCS) variables between obese (*n* = 30) and normal weight (*n* = 29) groups

Variables		Median nerve	Normal weight (*n* = 29) Median (Q1-Q3)	Obese (*n* = 30) Median (Q1-Q3)	p
Latency (ms)	Distal	Rt.	3.05 (2.65–3.67)	3.10 (3–3.3)	0.766
		Lt.	3.15 (2.82–3.57)	3.30 (2.9–3.5)	0.873
	Proximal	Rt.	7.30 (6.47–8.22)	7.15 (6.9–7.65)	0.543
		Lt.	7.35 (6.47–8.05)	7.40 (6.92–7.98)	0.885
Amplitude (mV)	Distal	Rt.	11.15 (8.54–12.8)	10.05 (8.33–11.80)	0.12
		Lt.	10.50 (8.79–13.17)	9.84 (9–11.45)	0.261
	Proximal	Rt.	**10.75 (8.71–12.2)**	**9.09 (7.62–10.20)**	**0.025**
		Lt.	10.30 (8.75–12.42)	8.74 (8.34–10.70)	0.073
Duration (ms)	Distal	Rt.	**10.00 (8.4–10.3)**	**10.50 (9.62–12)**	**0.020**
		Lt.	**10.00 (9.42–10.57)**	**10.70 (9.57–12.00)**	**0.047**
	Proximal	Rt.	**10.00 (9–10.57)**	**10.85 (10–11.88)**	**0.019**
		Lt.	10.05 (9.35–11)	11.00 (10–12)	0.050
Velocity (m/s)		Rt.	55.00 (53.27–58.10)	54.45 (51.12–57.4)	0.495
		Lt.	56.00 (51.12–60.25)	55.15 (51.47–58.2)	0.862

The bold values in the tables shows the data are statistically significant

**Table 5:** Comparison of tibial motor nerve conduction study (NCS) variables between obese (*n* = 30) and normal weight (*n* = 29) groups

Variables		Tibial nerve	Normal weight (*n* = 29) Median (Q1-Q3)	Obese (*n* = 30) Median (Q1-Q3)	p
Latency (ms)	Distal	Rt.	3.5 (3.1–4.3)	3.75 (3–4.25)	0.958
		Lt.	3.7 (3.15–4.2)	3.6 (3.32–4.1)	0.927
	Proximal	Rt.	12.45 (11.02–13.05)	12 (11.22–12.68)	0.549
		Lt.	12.3 (11.22–13.17)	12.20 (11.22–13.20)	0.756
Amplitude (mV)	Distal	Rt.	**15.60 (12.67–20.6)**	**13.05 (10.2–16.2)**	**0.027**
		Lt.	**17.00 (12.82–20.32)**	**13.6 (8.96–15.78)**	**0.009**
	Proximal	Rt.	**12.1 (10.55–15)**	**8.5 (7.04–11.18)**	**<0.001**
		Lt.	**13.05 (10.2–15.6)**	**9.08 (6.58–11.65)**	**0.002**
Duration (ms)	Distal	Rt.	**8.50 (7.92–10)**	**10.00 (9–11)**	**0.032**
		Lt.	8.9 (7.57–10)	9.5 (9–10.88)	0.218
	Proximal	Rt.	9.4 (8.3–10.15)	9.9 (9–11)	0.127
		Lt.	9.25 (7.82–10.5)	10.00 (8.925–10.5)	0.632
Velocity (m/s)		Rt.	40.10 (39.45–43.77)	39.60 (37.02–44.1)	0.524
		Lt.	40.75 (36.87–42.3)	39.90 (37.52–41.30)	0.627

The bold values in the tables shows the data are statistically significant

**Table 6:** Comparison of median sensory nerve conduction study (NCS) variables between obese (*n* = 30) and normal weight (*n* = 29) groups

Variables	Median nerve	Normal weight (*n* = 29) Median (Q1-Q3)	Obese (*n* = 30) Median (Q1-Q3)	p
Latency (ms)	Rt.	2.07 (1.86–2.34)	2.01 (1.81–2.27)	0.484
	Lt.	2.07 (1.92–2.28)	2.13 (1.92–2.45)	0.499
Amplitude (μV)	Rt.	**25.15 (19.42–34.07)**	**17.35 (15.2–26)**	**<0.001**
	Lt.	**25.15 (18.77–36.07)**	**18.75 (13.67–23.83)**	**0.009**
Duration (ms)	Rt.	**1.65 (1.38–1.8)**	**1.8 (1.72–2.1)**	**0.003**
	Lt.	**1.8 (1.5–1.87)**	**1.9 (1.8–2.1)**	**0.012**
Velocity (m/s)	Rt.	55.6 (47.75–61.92)	55.6 (51.15–60.53)	0.867
	Lt.	54.05 (50.6–62.15)	53.1 (47.45–58.8)	0.211

The bold values in the tables shows the data are statistically significant

**Table 7:** Comparison of sural sensory nerve conduction study (NCS) variables between obese (*n* = 30) and normal weight (*n* = 29) groups

Variables	Sural nerve	Normal weight (*n* = 29) Median (Q1-Q3)	Obese (*n* = 30) Median (Q1-Q3)	p
Latency (ms)	Rt.	2.28 (1.98–2.68)	2.19 (1.99–2.4)	0.222
	Lt.	2.46 (2.1–2.64)	2.22 (1.99–2.45)	0.089
Amplitude (μV)	Rt.	**22.75 (16.64–28.85)**	**16.55 (11.1–21.08)**	**0.004**
	Lt.	**23.45 (18.81–30.35)**	**12.9 (11.22–17.83)**	**<0.001**
Duration (ms)	Rt.	1.91 (1.8–2.15)	2.1 (1.8–2.4)	0.113
	Lt.	1.90 (1.8–2.1)	2.05 (1.8–2.4)	0.108
Velocity (m/s)	Rt.	48.9 (43.7–53.8)	49.25 (43.95–55.60)	0.716
	Lt.	46.4 (42–52.4)	47.7 (44.78–53.90)	0.682

The bold values in the tables shows the data are statistically significant

## Discussion

Obese persons had significantly higher body weight and BMI than normal weight controls. While comparing the nerve conduction study variables of peripheral somatic nerves, compound muscle action potential (CMAP) of median and tibial nerves showed lower amplitudes (median- proximal, and tibial- distal and proximal) in obese. Further, they had prolonged CMAP durations for median and tibial nerves. Similarly, the findings were found to be consistent with decreased sensory nerve action potential (SNAP) amplitudes and increased SNAP durations for median and sural sensory nerves.

The durations of the CMAPs and SNAPs mainly reflect the relative conduction rates of the impulses as they travel along the various axons between the stimulating and recording points [10]. In addition, CMAPs duration is primarily the measure of synchronized discharge of individual muscle fiber (i.e., the extent to which each of the individual muscle fibers fire at the same time). If there is a significant difference in the conduction velocity among nerve fibers, the duration will be prolonged. This is mainly related to the range of the conduction velocities of the large myelinated fibers. Furthermore, it is essential to note that latencies and conduction velocities reflect the fastest conducting fibres but, the many other slower conducting fibers participate in CMAP area and amplitude as well [11]. Increased CMAP (median and tibial nerves) and SNAP (median and sural nerves) durations in this study could be due to decreased conduction of nerve fibers (fastest and slower) in respective nerves resulting by either decreased myelination or axonal impairment (metabolic defect). However, there were no significant decrease in onset latencies and motor and sensory conduction velocities in obese persons. Thus, it can be concluded that increased CMAP duration could be due to alteration in slower conducting fibres. Our finding is supported by the study of Brismar et al.[12] and Selim et al. [13] that some nerve fibers might be more susceptible to damage than others, in particular, the small caliber or non-myelinated fibers, while others with diameters large enough to sustain the normal conduction velocities may be spared.

Whenever surface recording electrodes are used, amplitudes are semi-quantitative measures of the number of axons conducting impulses from the stimulating to the recording points. Amplitude (CMAP and SNAP) is a function of the total number of fibers stimulated and the synchronicity of the impulses [6]. There are several other influencing factors like the relative conduction rates along the axons, the distance between the recording electrodes and the fibers (nerve or muscle) generating the impulses. The CMAP amplitudes, in addition, are indicative of the efficiency of neuromuscular transmission, and the number of muscle fibers composing the recorded muscle that can generate action potentials [10, 14, 15].

This study revealed lower CMAP and SNAP amplitudes in obese as compared to normal weight controls. This could be due to variation in the relative conduction rates along the axons and decrease in the number of synchronically discharged neurons. Duration and amplitude are closely related: as the duration becomes more prolonged (i.e., the response becomes dispersed), the amplitude decreases [16]. Since, onset latencies and conduction velocities are normal, reduction in amplitudes also supports the probability of affecting slower and small calibre susceptible fibers. This finding is also supported by Ralph et al. [17], reporting that the sensory and mixed nerve amplitudes were lower in obese than in thin subjects. Similar results were obtained by Giacinta et al. [18], who found that the obese group showed significantly decreased compound muscle action potential amplitude of tibial and peroneal nerves and decreased sensory action potential amplitude of all nerves. They also aided the reason for decrement in amplitudes could be specific metabolic alterations [19] as in non-diabetic obese showing hyperinsulinemia and low insulin sensitivity that may be preclinical reason for onset of diabetes [20]. Similarly, a study by Robert Werner [21], noticed that obesity does not influence carpal canal pressure or the size of the median nerve at the wrist. However, there is a strong association between slowed median nerve conduction and increased nerve size, which suggests endoneural edema as a metabolic mechanism; the conduction slowing does not appear to be related to mechanical stress. Similar results were also obtained by other researchers [22–24], but they postulated the reason for decrease in amplitude could be a thicker subcutaneous adipose layer since most routine NCSs are performed using percutaneous stimulation and recording technique.

In this study, onset latencies, F-wave latencies and nerve conduction velocities did not differ significantly between the groups. Similar to this study, Buschbacher found no association between ulnar NCS and weight in ulnar nerve motor conduction to the abductor digiti minimi [25]. In contrast to this study, Dumitru mentioned that, since more adipose tissue in the epineurium would tend to better insulate the axon, the nerves of more obese persons might be expected to conduct their impulses more rapidly than in thin persons [26]. Simmons et al. [27], however, demonstrated that overweight patients had faster ulnar Across-elbow (AE) NCSs, and suggested that the skin measurements overestimate the nerve distance in this population leading to a falsely fast NCS. Similar to Simmons et al.[27], Landau et al. [28] reported that increasing BMI directly correlated with increasing ulnar motor NCV across the elbow but not with forearm NCV. Across-elbow (AE) ulnar motor NCS may be falsely increased in patients with a high BMI, probably due to distance measurement factors.

Obesity is well known risk factor for metabolic derangements, like functional alterations on different ion channels, especially on Na-K channels of nodes of Ranvier [21]. Thus, it may be concluded that prolonged CMAP and SNAP durations might be due to a specific metabolic alteration affecting mostly slower conducting fibers and decreased amplitudes of mixed and sensory nerves might be due to decreased axonal number stimulation or actual decrease in number of axonal fibers, or defect at NMJ in non-diabetic obese.

## Conclusion

This study, although conducted in a small number of patients and lacks the relation between body fat composition and Nerve conduction, documents subclinical peripheral nerve impairment in non-diabetic obese with abnormal NCS parameters. Prolonged durations but normal onset latencies and conduction velocities suggest involvement of slow conducting fibers. Further, reduced amplitudes might be due to decreased number of stimulated fibers or/actual decrease in number of axonal fibers or/defect at NMJ in non-diabetic obese. These changes could be due to metabolic alterations due to obesity. These abnormal somatic neural functions depicted by NCS strongly suggest that non-diabetic obesity could lead to future clinical neuropathy.

Further experimental studies based on a larger sample of obese patients, more accurate neurophysiological (i.e., near-nerve recording) and neuropathological (i.e., intraepidermal nerve fibers investigation) techniques, as well as animal models of obesity including histological findings, would provide stronger support for the effects of obesity on impaired somatic neural functions.

## Abbreviations

μV: Microvolt
BMI: Body mass index
BPKIHS: B. P. Koirala Institute of Health Sciences
CMAP: Compound muscle action potential
Ht: Height
Lt: Left
m/s: Meter per second
MNCS: Motor nerve conduction study
ms: Millisecond
mV: Millivolt
NCS: Nerve conduction study
Rt: Right
SNAP: Sensory nerve action potential
SNCV: Sensory nerve conduction study
WHO: World Health Organization
WHR: Waist-hip ratio
Wt: Weight
